# *In vitro* Assay of Human Gingival Scaffold in Differentiation of Rat’s Bone Marrow Mesenchymal Stem Cells to Keratinocystes

**Published:** 2012

**Authors:** Nasser Mahdavishahri, Maryam Moghatam Matin, Masoud Fereidoni, Zahra Yarjanli, Seyed Ali Banihashem Rad, Saeedeh Khajeh Ahmadi

**Affiliations:** 1 *Department of Biology, Faculty of Sciences, Ferdowsi University of Mashhad, Mashhad, Iran.*; 2 *Dental Research Centre, Faculty of Dentistry, Mashhad University of Medical Sciences, Mashhad, Iran*; 3 *Oral and Maxillofacial Diseases Research Centre, Faculty of Dentistry, Mashhad University of Medical Sciences, Mashhad, Iran*

**Keywords:** Acellular gingiva, Keratinocytes, Tissue engineering

## Abstract

**Objective(s):**

Tissue engineering is an attractive science because it promises new therapeutic strategies for repairing organs that have lost functions due to damage. The purpose of this study was to evaluate induction effect of human gingival scaffold in tissue engineering for skin regeneration.

**Materials and Methods:**

Tissue samples were obtained from crown-lengthening procedures and wisdom teeth removal. The samples were decellularized and used as a scaffold for loading of rat BM-MSCs. The human gingival scaffolds loaded by bone marrow mesenchymal stem cells were derived from Wistar rat. Finally, it was evaluated via electron micrographs, as well as immunohistochemical techniques at day 7, 14, and 28 after initial seeding.

**Results:**

The histologic sections of human gingival scaffold –loaded rat BM-MSCs demonstrated formation of epithelial like layers at days 7, 14 and 28 after initial seeding. Scanning electron microscope (SEM) of the scaffolds indicated formed desmosomal adhesions, which revealed a degree of differentiation toward keratinocytes. The results of immunohistochemical staining were strongly positive for multi cytokeratin (CK) 14 days after initial seeding in epithelial differentiation. Rat BM-MSCs which loaded on human gingival scaffold is capable of differentiating toward keratinocytes.

**Conclusion:**

Gingival tissues were presented as a natural scaffold for attachment and differentiation of bone marrow mesenchymal stem cells towards keratinocytes, and might be used as suitable scaffold for reconstruction of the skin.

## Introduction

Mesenchymal stem cells (MSCs) have a capacity of self renewal and multi-lineage differentiation, for tissue engineering (1). MSCs are used in tissue engineering for regenerating tissues (2, 3). The skin consists of two separate tissue components: a covering epithelium and an underlying connective tissue. As a surface lining, the skin protects deeper organs from microorganisms and foreign body agents (4). Skin regenerative therapies which replace defective skin using autologous explants are being investigated using current understandings of stem cell biology, and regenerative medicine (5-9). The keratinocytes at the wound margin migrated across the defect in epithelial regeneration (10).

The majority of changes involved in cell maturation are the change in cell size and shape which are accompanied by a synthesis of more structural proteins in the form of tonofilaments, the appearance of new organelles, and the production of additional intracellular material (11). In addition, membranes containing organelles, like keratohyaline granules, Odland bodies, and so on, appear inside the cell and intercellular desmosomal ligands construct in late stages of differentiation (12). In a number of clinical trials, MSCs were used for tissue regeneration in spinal cord injuries, ischemic heart lesions, bone defects, skin and neurodegenerative diseases. In a recent study, BM- MSCs are used for skin repair of diabetic and non-diabetic mice (9). Connective tissue adjacent to the epithelia had induction effect on 

epithelial formation (13). In this study we used decellularized human gingiva for differentiation of BM- MSCs towards epithelial cells.

## Materials and Methods

**Figure 1:**
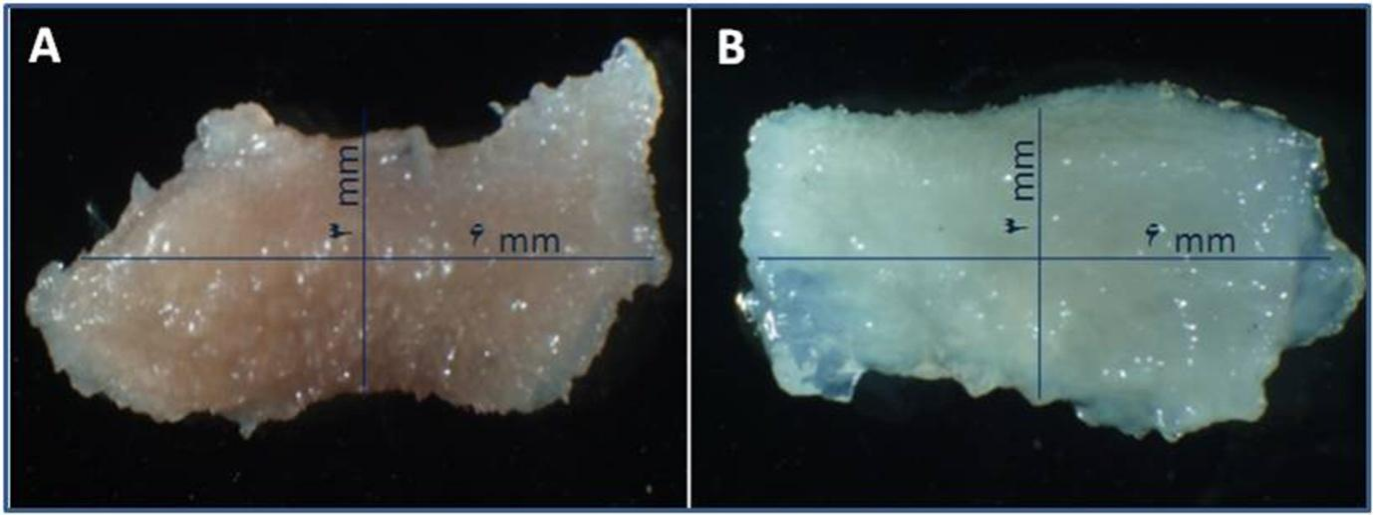
Clinically, A, This appeared as human gingiva **.B**, Decellularized human gingiva as scaffold for tissue-engineering

Ten gingival samples were taken by a periodontist in accordance with professional ethics. Samples were taken from 20-45 years old humans. The patients were healthy without any diseases, were not taking any medication and were nonsmokers. Tissue samples were obtained from crown-lengthening procedures and wisdom teeth removal that were maintained in normal saline. After tissue preparation, the sample size reached to 3×6 mm (Figure1, A) Five tissue samples were prepared for decellularization of tissues, we preformed slow and fast freezing via liquid nitrogen, and immersed them in 1% SDS for 24 hr and then stored them in1% Triton for 12 hr.

Sodium dodecyl sulfate (SDS) (CinnaGen, Tehran, Iran) in phosphate buffered salin (PBS) was added to the specimens for 24 hr. 1% Triton X-100 (CinnaGen, Tehran, Iran) solution in PBS (CinnaGen, Tehran, Iran) was added to the mixture for 12 hr (manual mixer in room temperature). The specimen was placed in PBS for 2 hr (14). Scaffolds were placed in 70% ethanol for sterilization for 30 min at 37^o^C. This procedure was carried out under a laminar hood (Pars Pajouhesh, Iran). Finally, scaffolds were washed with sterile distilled water; afterwards they were immersed in a sterile PBS solution for one hr (Figure 1, B).

In order to prepare bone marrow mesenchymal stem cells (BM-MSCs), male adult Wistar rats were anesthetized with chloroform and their femoral bone was removed under sterile conditions. First, the two ends of the bone were removed and bone marrow contents were extracted and cultured with Dulbecco’s Modified Eagle's Medium (DMEM) (GIBCO/USA) supplemented with 15% to 20% fetal bovine serum (GIBCO/USA) and 10 µl penicillin/streptomycin (Biosera, UK). The resulting suspension was transferred to a flask (Orange Scientific, Belgium) and incubated at 37°C in 5% CO_2_ (Lab-Line, USA). After 24 hr, the overlying solution was removed and the adhered cells of the bottom of the flask remained. 

**Figure 2:**
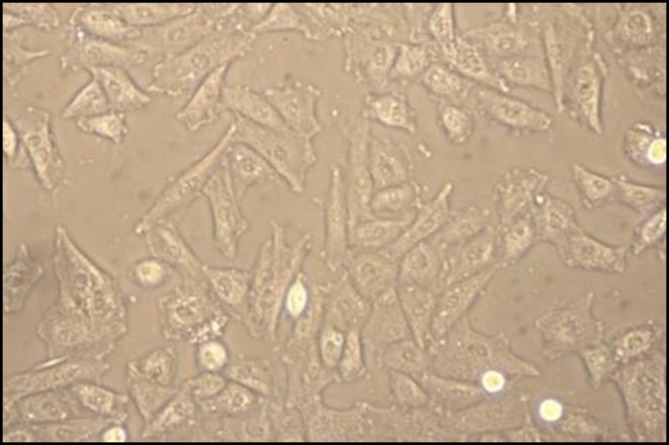
Isolation of BM-MSCs at passage 4 (×400)

After the last passage, we added trypsin in order to release the cells adhered to the flask. Then flask contents were transferred to a falcon tube (Orange Scientific, Belgium). After centrifugation and determination of the cell count with a Neobar slide, cell suspensions were prepared with a concentration of 2×10^5^ cells/cm^2^. To assess colony-forming efficiency, fourth passage cells were fixed with 4% formalin and then stained with 0.1% toluidine blue (Figure 2).

The scaffolds were transferred to the 24 wells plate (Orange Scientific, Belgium). In each well scaffold seeded with 2×10^5 ^cells/cm^2^ in Dulbecco's Modified Eagle's Medium and incubated at 37°C in 5% CO_2_ (direct method). Five scaffolds were used as a control medium culture. Medium was replaced every 3 days.

The samples were fixed with Bouin's fixator, and then stained with hematoxilin-eosin (H&E), hematoxilin weigert -peak indigo carmine (H&P), and carmine (C.I.75470). Immunohistochemical staining with Multi cytokeratin (CK) (Catologue No. AE1, CloneAE3; Novocastra Laboratories Ltd, Newcastle, U.K.) , was also done. The samples were examined under a light microscope (Olympus, Japan: IX70), Transmission electron microscope (TEM) (Leo–910, Germany), and scanning electron microscope (SEM) (Leo-VP1450, Germany) at days 7, 14, and 28 after initial seeding. 

In order to prepare electron microscope imaging, specimens were fixed with 2.5% gluteraldehyde and 0.1 molar sodium cacodilate 

for 24 hr, after being washed with normal saline, (TAAB, UK). Scaffolds were dehydrated with incremental concentrations of 30%, 50%, 70%, 90%, and 100% ethanol. After dehydration; specimens were placed on grids and coated with palladium-Au. Specimens were observed under a SEM (Leo-VP1450, Germany).

**Figure 3:**
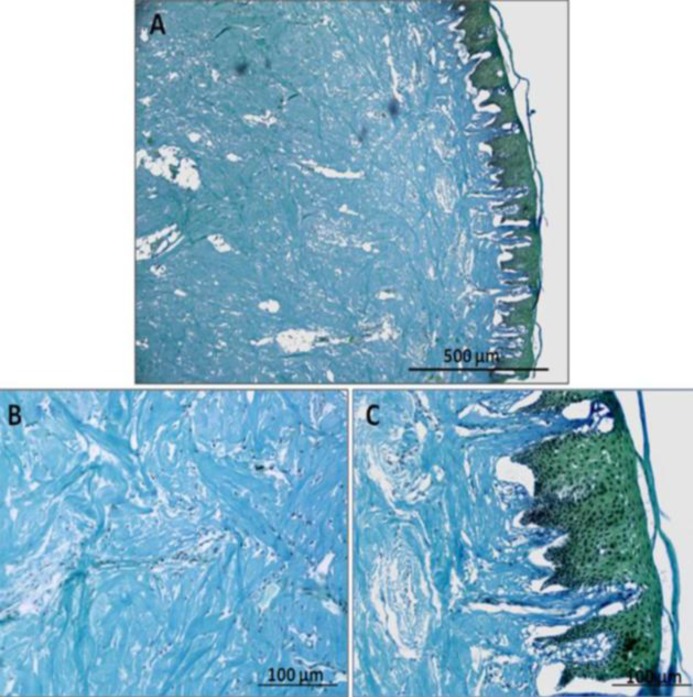
A, B and C, Section through the human gingival tissue which are stained by the H&P to demonstrate epithelium layer and collagen fiber in the lamina propria

**Figure 4:**
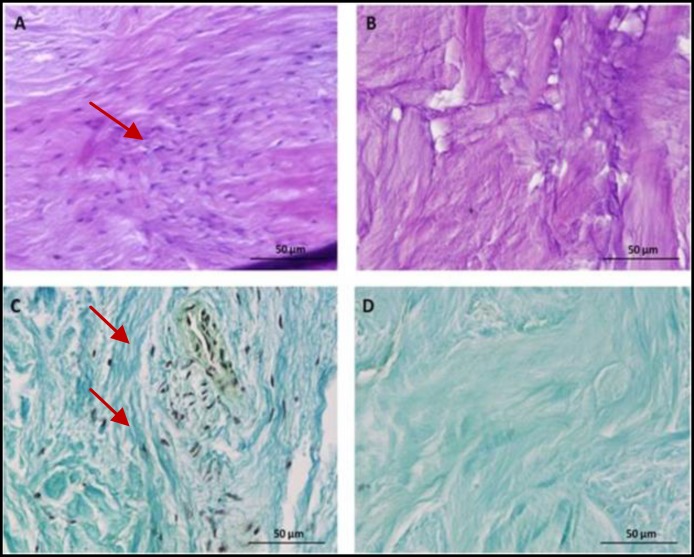
A and C, human gingival tissue (H&E staining). B and D, decellularization human gingival tissue (H&P staining) (Red arrows point to the cells)

**Figure 5:**
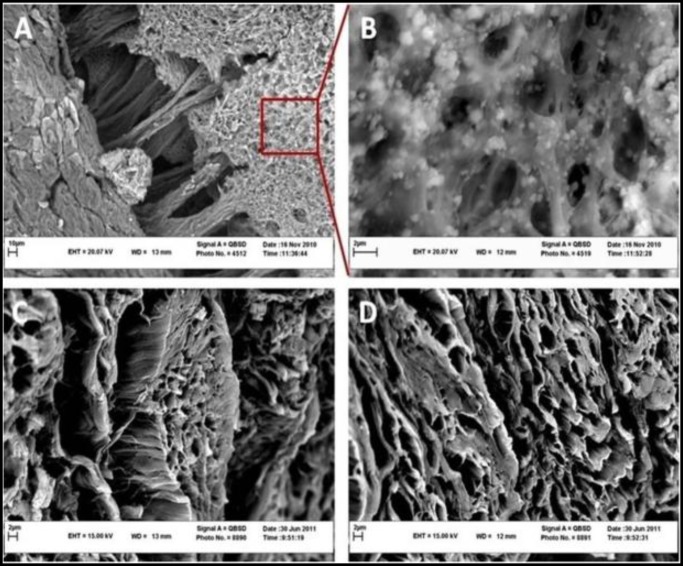
Scanning electron micrographs of the decellularized human gingival tissue. A and B, overall structure of human gingival preserved after decellularization. C and D, collagen fibers in the connective tissue were intact

**Figure 6:**
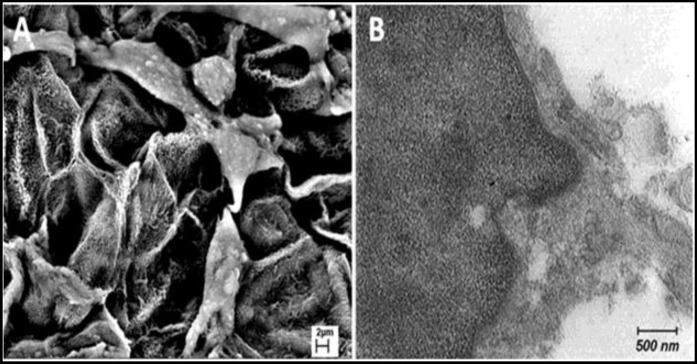
The appearance of cell pseudopod formation with scanning **(A)** and transmission **(B)** electron microscope at day 7 after initial seeding (stars depict cell pseudopods)

**Figure 7:**
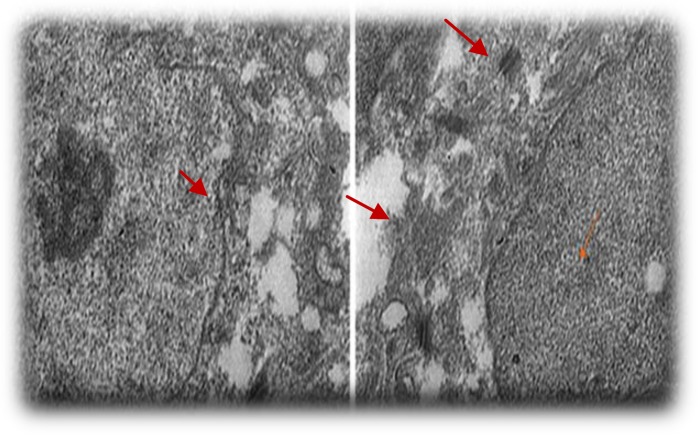
Intracellular junction**. **A and B, Transmission electron micrographs of cell from the gingival epithelium. The cell has desmosome attachment to adjacent cells at day 14 after initial seeding

**Figure 8:**
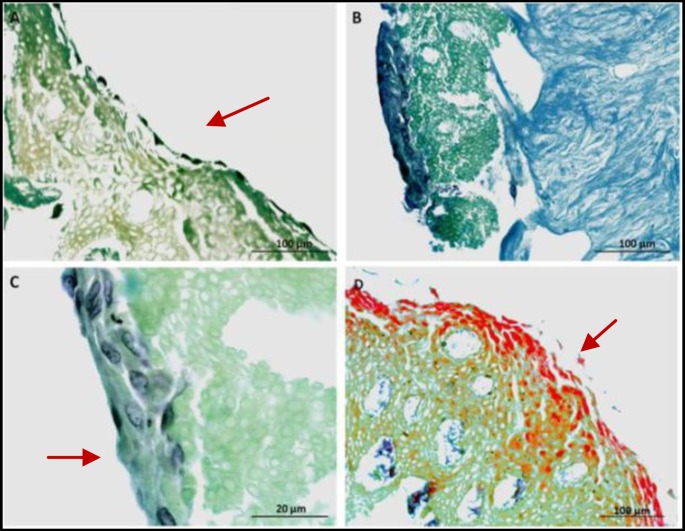
A, B and C, the histologic section of human gingival scaffold –loaded rat BM-MSCs demonstrated formation of epithelial-like layer at days 7, 14 after initial seeding (H&P staining). D, epithelial like layer formation at day 28 after initial seeding (Carmine staining)

**Figure 9:**
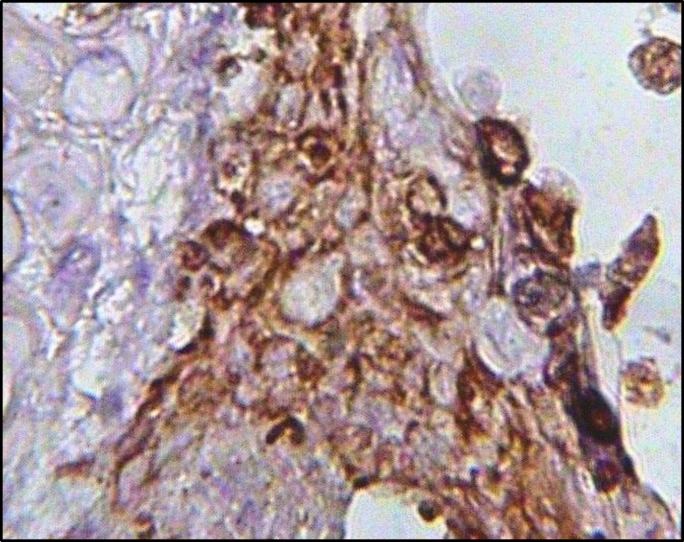
Immunohistochemical staining for multi cytokeratin (CK) showed positive cell staining (brown) in epithelial differentiation at days 14 after initial seeding (×400)

For TEM evaluation, specimens were fixed with gluteraldehyde and 1% osmium tetroxide and dehydrated with incremental concentrations of ethanol. Specimens were placed in propylene oxide for 30 min, and finally placed in pure resin for 30 min (ARALDITE 502 Resin Kit) (TAAB, UK). The specimens were segmented into a thickness of 80 nm with ultra-microtome (LKB, UK). These sections were studied and photographed with a TEM (Leo–910, Germany).

## Results

Human gingiva is composed of epithelium and connective tissue (Figure 3). These tissues were decellularized and prepared for use as scaffold for bone marrow mesenchymal stem cells derived from Wistar rat. 

As shown in Figure 4, cells are omitted from specimens during the decellularizing procedure.

Scanning electron micrographs of the decellularized human gingival tissue showed that the overall structure of human gingiva is preserved after decellularization (Figure 5). Transmission electron microscope and Scanning electron microscope images showed cell pseudopod formation at day 7 after initial seeding (Figure 6). Transmission electron micrographs of cells from the gingival epithelium revealed desmosome attachment to adjacent cells at day 14 after initial seeding (Figure 7). The histologic section of human gingival scaffold loaded rat BM-MSCs demonstrated formation of epithelial like layer at days 7, 14 and 28 after initial seeding (Figure 8). The results of immunohistochemical staining were strongly positive for multi cytokeratin (CK) at days 14 after initial seeding in epithelial differentiation (Figure 9). Therefore rat BM-MSCs which are loaded on human gingival scaffold are capable of differentiating toward keratinocytes.

## Discussion

Tissue engineering methods uses scaffolds and stem cells to repair tissues and stimulate organ function. Decellularized matrixes of cartilage, bone, urethra, umbilical cord, and dermis were used as scaffolds in tissue engineering (15-20) Collagen hydrogels were also used as scaffold for dermal regeneration, but satisfactory results were not obtained (7). 

In this study, human gingival scaffold has been used for rat BM-MSCs differentiation towards keratinocytes. The human gingival epithelium is a stratified squamous epithelium consisting of cells tightly attached to each other and arranged in a number of distinct layers (21).

Wu *et al* showed that BM-MSCs could be useful in treating diabetic and non-diabetic ulcers by increasing epithelial renewal, cell penetration and angiogenesis. The rat BM-MSCs which was transplanted to the skin ulcers, accelerated wound healing and epithelium regeneration (9). Transmission electron micrographs showed intracellular junctions. The cell has desmosome attachment to adjacent cells at day 14 after initial seeding (Figure 7). The rat BM-MSCs were loaded on human gingival scaffolds for epithelial formation (Figure 8). Cytokeratines are a family of interacellular fibrous proteins that represent an excellent marker for epithelial differentiation (22). In the present study, immunohistochemical staining showed positive expression for multi cytokeratin in epithelial differentiation (Figure 9). So, BM-MSCs can differentiate toward keratinocytes by induction effect of human gingival scaffold.

## Conclusions

Reconstruction of the skin is one of the major goals in dermal technology. Human gingival scaffold is suitable for tissue engineering. If the hypothesis discussed here is true, gingival tissue as a natural scaffold can be used for attachment and differentiation of bone marrow mesenchymal stem cells toward keratinocytes and might be used as a suitable scaffold for reconstruction of the skin. 
